# Potential adverse events in Japanese women who received tozinameran (BNT162b2, Pfizer-BioNTech)

**DOI:** 10.1186/s40545-021-00326-7

**Published:** 2021-05-31

**Authors:** Rumiko Shimazawa, Masayuki Ikeda

**Affiliations:** 1 Department of Clinical Pharmacology, Tokai University School of Medicine, Isehara, Kanagawa 259-1193 Japan; 2 Department of Medical Informatics, Kagawa University Hospital, Miki-cho, Kagawa 761-0793 Japan

## Abstract

Reports of cerebral venous sinus thrombosis and intracranial hemorrhage (ICH) following the administration of coronavirus vaccines have raised concerns regarding their safety. Although no regulatory authority has recognized ICH as an adverse event associated with tozinameran (BNT162b2, Pfizer-BioNTech), fatal and non-fatal cases have been reported. In Japan, 10 fatal cases (five men and women) have been reported to date. Four of the five women died of ICH and the other died of aspiration pneumonia, whereas all five men died of causes other than stroke. This imbalance is incompatible with the mortality data on cardiovascular diseases in the National Statistics, which show no apparent disparity between sexes or between hemorrhagic and ischemic stroke. Cumulatively, our analysis reveals a disproportionately high incidence of death by ICH in Japanese women who received tozinameran, suggesting a potential association of ICH with the vaccine. Although we understand that the benefits of tozinameran still outweigh the risks, we believe that a causal link with the vaccine is not proven but possible and warrants further analysis.

## Introduction

Recent experience with ChAdOx1 nCoV-19 [1] has demonstrated that pharmacovigilance is essential for the early detection of signs of severe adverse events. Reports of immune thrombocytopenia (ITP) following the administration of vaccines against severe acute respiratory syndrome coronavirus 2 (SARS-CoV-2) have raised concerns regarding their safety [1–5]. Cerebral venous sinus thrombosis (CVST) resulting from ITP is a neurovascular emergency typically affecting young adults and is more common in women than in men. CVST often results in a potentially fatal intracranial hemorrhage (ICH; intracerebral and/or subarachnoid hemorrhage) [6]. Although no regulatory authority has recognized ICH as an adverse event associated with tozinameran (BNT162b2, Pfizer-BioNTech), fatal and non-fatal cases have been reported [4, 5]. Autoimmune mechanisms common to Covid genetic vaccines [1, 2] are proposed to induce ITP and internal bleeding.

## Disproportionately high incidence of death by ICH in Japanese women who received tozinameran

The Ministry of Health, Labour and Welfare (MHLW) initiated tozinameran vaccinations on February 17, 2021 for healthcare workers. Vaccines other than tozinameran remain unapproved and unavailable in Japan. As of April 18, 2021, an estimated 1.21 million first and 0.72 million second doses of tozinameran have been administered. The MHLW has reported 10 fatal cases (five men and five women) to date [7]. Four of these 10 cases died of ICH; all of these were women who died after the first shot. No fatal case with ischemic stroke has been reported. The remaining six cases comprised five men and one woman. The woman died of aspiration pneumonia 4 days after the first shot. The five men died of causes other than stroke, i.e., acute heart failure, drowning, ventricular fibrillation, sepsis, and cardiopulmonary arrest of unknown origin. Here, we describe the histories of the four ICH cases listed on the MHLW website [7]. In three of the four cases, the clinical course and unwitnessed death of a previously healthy woman with unremarkable history or risk factors of cerebrovascular diseases were atypical of the common types of cerebral hemorrhage, e.g., putaminal, thalamic, and cerebellar. No platelet count or other test data were available for three of the four cases because they were found dead at home. Details of the postmortem examination, i.e., imaging and autopsy, were also unavailable.

*Case 1* was a 61-year-old woman with no significant history. She was found dead at home by her husband 3 days after receiving the first shot of tozinameran, with no episode reported in the intervening time. A spinal tap revealed bloody cerebrospinal fluid. Neither autopsy nor postmortem imaging study was performed.

*Case 2* was a 26-year-old woman with no underlying conditions. After the first shot of tozinameran, her subsequent course was unremarkable until she was found dead 4 days later at home. Postmortem imaging revealed a hematoma 3.5 cm in diameter at the left cerebellopontine angle compressing the brainstem and secondary subarachnoid hemorrhage.

*Case 3* was a 72-year-old woman with hepatitis C and dyslipidemia. Three days after the first shot of tozinameran, she developed dysarthria with complaints of headache and nausea. Brain imaging revealed a large hematoma with ventricular rupture. Her platelet count was 216,000/mm^3^. She died 5 days after receiving tozinameran.

*Case 4* was a 69-year-old woman. No underlying condition was specified. She had been well until she was found dead at home 9 days after the first shot of tozinameran. An autopsy revealed that she died of ICH. No other information was available.

*Case 5* was a 102-year-old woman with chronic heart failure. Ten days before the first shot, she had developed aspiration pneumonia, which was empirically treated with clarithromycin. She died 4 days after receiving tozinameran. Thrombocytopenia was not reported. The cause of death was considered to be aspiration pneumonia, which was revealed by computed tomography. No autopsy was performed.

*Case 6* was a 65-year-old man with an unremarkable history. He was reported to have been well at 18 days after the first shot. He was found dead at home 3 days later when a policeman, informed of his absence from the office, visited to ask for him. Postmortem inspection showed blood coagula in the oral cavity. He was presumed to have died of acute heart failure on the grounds that his living conditions suggested alcoholism and heavy smoking. No autopsy or other postmortem examination was performed.

*Case 7* was a 62-year-old man with hypertension, diabetes, and obesity. He was reported to have taken an unspecified anti-thrombotic drug. On the day after the second shot of tozinameran, he was found dead in the bathtub by a housemate. Autopsy of the lungs revealed the cause of death to be drowning without ICH or any other significant pathology.

*Case 8* was a 51-year-old man with no underlying conditions. He was found apneic in bed at midnight 14 days after the first shot. He was transferred to a hospital, but resuscitation failed. His housemate was informed that he had died of ventricular fibrillation. No autopsy or other postmortem examination was performed.

*Case 9* was a 73-year-old man with chronic renal failure. He had been on hemodialysis for 6 months before the second shot. In the night after the shot, he became febrile with vascular access infections and purulent vertebral osteomyelitis. Thrombocytopenia was not reported. He died of septic shock 8 days later. No autopsy was performed.

*Case 10* was a 37-year-old man. He was reported to have had a history of unspecified arrhythmia, electrocardiographic anomaly, and hay fever. He had been well until he was found dead in bed in the morning 3 days after the second shot. No autopsy or other postmortem examination was performed.

## Sex imbalance in ICH incidence

The MHLW concluded that these data did not show a link between tozinameran vaccination and the deaths because ICH can occur naturally and is happening more frequently in the general population than in the vaccinated population [8]. However, this conclusion could be misleading when the risk of side effects is as low as 3.9 in every million doses [3]. To detect remarkable signals with higher sensitivity, we focused on the sex imbalance in ICH incidence. Four of the five deceased women died of ICH and the other died of aspiration pneumonia, whereas all five men died of causes other than stroke. This imbalance is incompatible with the mortality data on cardiovascular diseases in the National Statistics [9], which show no apparent disparity between sexes. First, the death rate by ICH (363/million) is 25% lower than that by ischemic stroke (486/million). Second, the death rate by ICH is comparable between the sexes (371/million in men and 355/million in women). Third, although the death rate by heart disease (1622/million in men and 1728/million in women) is > 4 times higher in both sexes than that by ICH, three men but no women died of heart disease after the administration of tozinameran. Cumulatively, our analysis reveals a disproportionately high incidence of death by ICH in women who received tozinameran in Japan.

## Imminent concern

The imminent concern would be ITP and CVST, which were observed in 19 patients (8 men and 11 women) who received the Pfizer vaccine in the USA [4]. On the basis of data from the UK, there is no evidence that women have higher mortality from ITP than men [3]. The lack of information essential for the diagnosis does not allow us any presumption of the cause of the disproportionately high incidence of death by ICH in women who received tozinameran in Japan. Reinforcement of the Japanese pharmacovigilance of SARS-CoV-2 vaccination is urgently required. We appeal to the MHLW to issue a caution notice to the public and healthcare professionals and to support them in monitoring thrombotic events more closely with appropriate feedback. In particular, the MHLW should list ITP-related symptoms for which vaccinated individuals should seek urgent medical consultation. Next, the ministry should assure continual monitoring of the situation and further review of ITP and ICH associated with tozinameran. During such pharmacovigilance, the early signs of rare side effects require intensive scientific investigations and clinical appraisal to exclude a causal link.

## Conclusions

Regulatory authorities are responsible for warning the public regarding the side effects of vaccines. Post-approval safety evaluation of new interventions such as SARS-CoV-2 vaccines is key to the identification of their optimal benefit–risk balance. Although we understand that the benefits of tozinameran still outweigh the risks, we believe that a causal link with the vaccine is not proven but possible and warrants further analysis.

## Data Availability

All data generated or analyzed during this study are included in this published article.

## Abbreviations

CVST: Cerebral venous sinus thrombosis
ICH: Intracranial hemorrhage
ITP: Immune thrombocytopenia
MHLW: Ministry of Health, Labour and Welfare
SARS-CoV-2: Severe acute respiratory syndrome coronavirus 2
